# 6α-Acetoxy­gedunin

**DOI:** 10.1107/S1600536809027998

**Published:** 2009-07-22

**Authors:** Margit Hofer, Harald Greger, Kurt Mereiter

**Affiliations:** a Comparative and Ecological Phytochemistry, University of Vienna, Rennweg 14, A-1030 Vienna, Austria; b Institute of Chemical Technologies and Analytics, Vienna University of Technology, Getreidemarkt 9/164SC, A-1060 Vienna, Austria

## Abstract

The title compound [systematic name: (1*S*,3a*S*,4a*R*,4b*S*,5*S*,6*R*,6a*R*,10a*R*,10b*R*,12a*S*)-5,6-bis­(acet­yloxy)-1-(3-fur­yl)-1,5,6,6a,7,10a,10b,11,12,12a-deca­hydro-4b,7,7,10a,12a-penta­methyl­oxireno[*c*]phenanthro[1,2-*d*]pyran-3,8(3a*H*,4b*H*)-dione], C_30_H_36_O_9_, is a limonoid-type triterpene isolated from *Aglaia elaeagnoidea* (A. Juss.) Benth. (Meliaceae) from Queensland, northern Australia. It contains the gedunin core of four *trans*-fused six-membered rings with an oxirane ring annelated to the fourth ring. A terminal 3-furyl unity and two acet­oxy groups in a mutual *cis*-disposition supplement the mol­ecule. A comparison between the gedunin cores of the title compound, the parent compound gedunin, and three further gedunin derivatives revealed considerable variations in their conformation stemming from the conformational lability of the first screw-boat ring and the third twist-boat ring. A sensitive measure for the third ring is one C—C—C—C torsion angle, which is 14.2 (2)° in the title compound, but varies in other cases from *ca* 20 to *ca* −40°. In the crystalline state, 6α-acetoxy­gedunin shows ten comparatively weak C—H⋯O inter­actions, with H⋯O distances in the range of 2.33–2.69 Å.

## Related literature

For general background to the genus Aglaia and its potential bioctivity, see: Brader *et al.* (1998 ▶); Engelmeier *et al.* (2000 ▶); Fuzzati *et al.* (1996 ▶); Greger *et al.* (2000 ▶, 2001 ▶); Hausott *et al.* (2004 ▶); Jimenez *et al.* (1998 ▶); Lavie *et al.* (1972 ▶). For related structures, see: Mitsui *et al.* (2006 ▶); Sutherland *et al.* (1962 ▶); Toscano *et al.* (1996 ▶); Waratchareeyakul *et al.* (2004 ▶). For the NMR spectra of related compounds, see: Connolly *et al.* (1966 ▶); Mitsui *et al.* (2006 ▶); Taylor (1974 ▶); Waratchareeyakul *et al.* (2004 ▶).
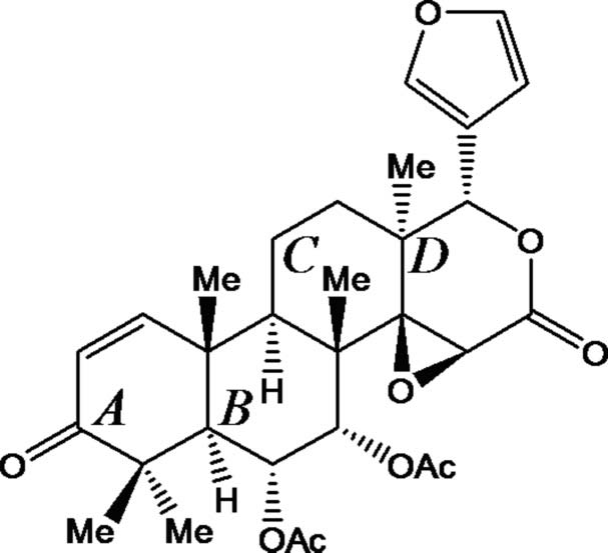


## Experimental

### 

#### Crystal data

C_30_H_36_O_9_
*M* *_r_* = 540.59
Orthorhombic, 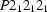

*a* = 6.475 (2) Å
*b* = 14.914 (5) Å
*c* = 28.713 (9) Å
*V* = 2772.8 (15) Å^3^
*Z* = 4
Mo *K*α radiation
μ = 0.10 mm^−1^
*T* = 173 K
0.62 × 0.40 × 0.25 mm

#### Data collection

Bruker SMART APEX CCD diffractometer
Absorption correction: multi-scan (*SADABS*; Bruker, 2003 ▶) *T*_min_ = 0.89, *T*_max_ = 0.98
39048 measured reflections
4509 independent reflections
4077 reflections with *I* > 2σ(*I*)
*R*_int_ = 0.031

#### Refinement

*R*[*F* ^2^ > 2σ(*F* ^2^)] = 0.037
*wR*(*F* ^2^) = 0.099
*S* = 1.04
4509 reflections
359 parameters
H-atom parameters constrained
Δρ_max_ = 0.26 e Å^−3^
Δρ_min_ = −0.24 e Å^−3^

### 

Data collection: *SMART* (Bruker, 2003 ▶); cell refinement: *SAINT* (Bruker, 2003 ▶); data reduction: *SAINT*, *SADABS* and *XPREP* (Bruker, 2003 ▶); program(s) used to solve structure: *SHELXS97* (Sheldrick, 2008 ▶); program(s) used to refine structure: *SHELXL97* (Sheldrick, 2008 ▶); molecular graphics: *SHELXTL* (Sheldrick, 2008 ▶); software used to prepare material for publication: *SHELXTL*.

## Supplementary Material

Crystal structure: contains datablocks global, I. DOI: 10.1107/S1600536809027998/fj2239sup1.cif

Structure factors: contains datablocks I. DOI: 10.1107/S1600536809027998/fj2239Isup2.hkl

Additional supplementary materials:  crystallographic information; 3D view; checkCIF report

## Figures and Tables

**Table 1:** Hydrogen-bond geometry (Å, °)

*D*—H⋯*A*	*D*—H	H⋯*A*	*D*⋯*A*	*D*—H⋯*A*
C11—H11*B*⋯O1^i^	0.99	2.59	3.410 (3)	141
C18—H18*C*⋯O1^i^	0.98	2.68	3.571 (2)	152
C18—H18*B*⋯O6^ii^	0.98	2.56	3.497 (2)	160
C22—H22⋯O6^ii^	0.95	2.69	3.601 (3)	162
C24—H24*B*⋯O2	0.98	2.40	3.066 (3)	125
C25—H25*C*⋯O2	0.98	2.50	3.112 (2)	121
C5—H5⋯O3	1.00	2.50	2.931 (2)	105
C9—H9⋯O3	1.00	2.55	2.944 (2)	103
C18—H18*B*⋯O5	0.98	2.45	2.934 (2)	110
C7—H7⋯O9	1.00	2.33	2.735 (2)	103

Symmetry codes: (i) 
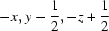
; (ii) 
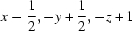
.

**Fig. 1.**
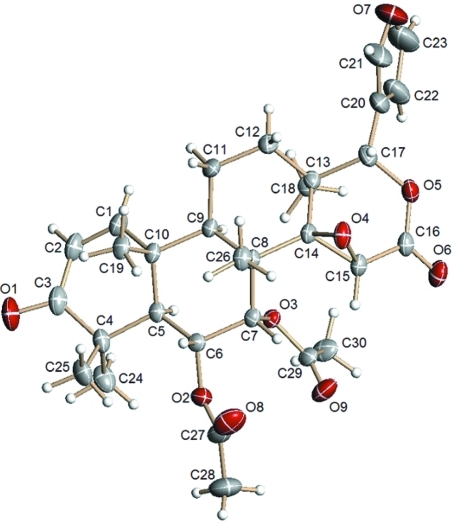
The structure of the title compound, showing 50% probability displacement ellipsoids and the atom-numbering scheme.

**Fig. 2.**
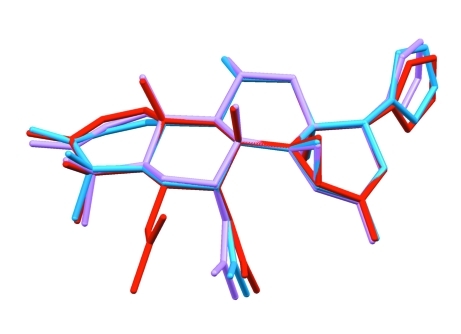
Superposition plot of the title compound (red) and 11α-hydroxygedunin (Mitsui et al., 2006; two independent molecules in blue and pink) after least squares fit of the A/B/C/D rings.

**Fig. 3.**
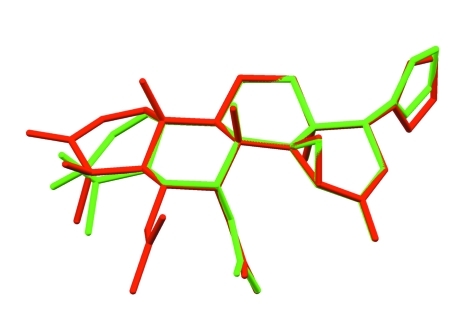
Superposition plot of the title compound (red) and gedunin (Toscano et al., 1996; green) after least squares fit of the B/C/D rings. The distance between the two terminal keto oxygen atoms O(1) (leftmost red and green atom) is 2.01 Å.

**Fig. 4.**
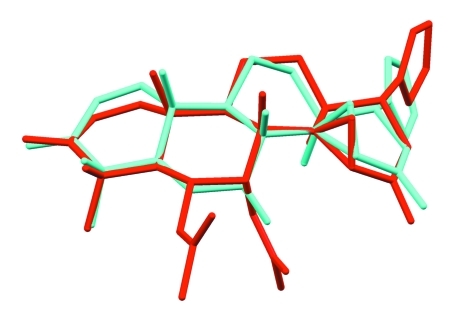
Superposition plot the title compound (red) and 7-oxogedunin (Waratchareeyakul et al., 2004; cyan) after least squares fit of the A/B/C/D rings. Note the difference in torsion angle T2 = C9—C11—C12—C13 (upper part of ring C), which is +14.2 (2)° in 6α-acetoxygedunin and -38.9° in 7-oxogedunin.

**Fig. 5.**
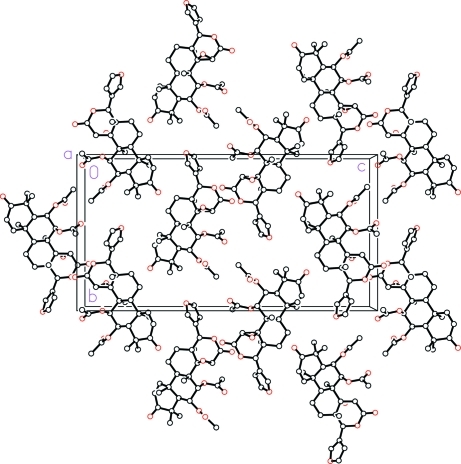
Packing diagram of 6α-acetoxygedunin viewed down the a axis.

## supplementary crystallographic information

### Comment

The genus Aglaia of the family Meliaceae has received scientific attention due to its bioactivity potential. Besides its unique chemical capacity to produce flavaglines with pronounced insecticidal, antifungal, and anticancer activities (Greger *et al.*, 2001; Engelmeier *et al.*, 2000; Hausott *et al.*, 2004), Aglaia is also characterized by the accumulation of bisamides, lignans, and triterpenes (Greger *et al.*, 2000). However, in contrast to other genera of that family the highly active triterpenoid limonoids appear to be rare in Aglaia only known so far from *Aglaia elaeagnoidea* collected in Sempu Island, Java, Indonesia (6α,11β-diacetoxygedunin; Fuzzati *et al.*, 1996), but not from samples collected in India and Thailand (Brader *et al.*, 1998). Our investigation of the root bark of *Aglaia elaeagnoidea* originating from northern Australia led now to the second isolation of a limonoid from this genus, namely the title compound 6α-acetoxygedunin. This compound was previously isolated from several other genera of the Meliaceae family, such as *Guarea grandiflora* Decne.ex Steud. (Jimenez *et al.*, 1998) or *Carapa guianensis* Aubl. (Lavie *et al.*, 1972), but its crystal structure has not been determined as yet.

6α-Acetoxygedunin contains the gedunin skeleton with four six-membered rings (A, B, C, D), which are all *trans*-fused and adopt screw-boat, chair, twist-boat, and twisted half-chair conformation, respectively (Fig. 1). The D-ring, a lactone, is stiffened by fusion with an oxiran ring and bears in equatorial position a furan ring in approximately perpendicular orientation to the main plane of the molecule. Bond length and angles are normal (*cf.* geometric parameters) and compare well with the parent compound gedunin (Toscano *et al.*, 1996), which is devoid of the 6-acetoxy group O2—(C27O8)—C28H_3_, and with three more gedunin derivatives, 11α-hydroxygedunin (Mitsui *et al.*, 2006), 11β-hydroxygedunin (Mitsui *et al.*, 2006), and 7-oxogedunin (Waratchareeyakul *et al.*, 2004; 7-acetoxy group replaced by a carbonyl oxygen with concomitant change of C7 from *sp*^3^ to *sp*^2^ hybridization). However, the torsion angles within the A/B/C/D rings of these five compounds show in part considerable variations and consequently the molecular conformations as well. This is visualized by three superposition plots of 6α-acetoxygedunin and its congeners shown in Figures 2 to 4. Fig. 2 demonstrates that 6α-acetoxygedunin and 11α-hydroxygedunin (Mitsui *et al.* 2006; it contains two independent molecules) have relatively closely matching conformations of their A/B/C/D rings. Fig. 3 compares 6α-acetoxygedunin and gedunin showing that their B, C, and D rings match very well, but that the A-rings display a significant mismatch. The torsion angle T1 = C3—C4—C5—C10 may be used as a qualitative measure for this match/mismatch: It is 30.7 (2)° in 6α-acetoxygedunin and 51.2° in gedunin, while the remaining three gedunin-type compounds have T1 angles between 32.2° (11α-hydroxygedunin) and 45.3° (7-oxogedunin). Fig. 4 demonstrates that the most outstanding difference in conformation exists between 6α-acetoxygedunin and 7-oxogedunin. This difference does not arise from the unlike hybridization of C7 (*sp*^3^ in title compound and *sp*^2^ in 7-oxogedunin), but is clearly provoked by ring C, which switches from a twist-boat conformation in 6α-acetoxygedunin *via* a virtual boat-intermediate into a twist-boat conformation of opposite twist in 7-oxogedunin. This can be tracked by the torsion angle T2 = C9—C11—C12—C13, which is +14.2 (2)° in 6α-acetoxygedunin (+21.6° in gedunin; +9.5° and +14.5° for the two independent molecules in 11α-hydroxygedunin), while it is -38.9° in 7-oxogedunin and -20.7° in 11β-hydroxygedunin. The described variations come essentially from the fact that B– and D-rings behave hard (B-ring in a relaxed and essentially invariant chair-conformation, D-ring in a twisted half-chair conformation fixed by oxiran ring and lacton group) while A-rings (screw-boat) and C-rings (between boat and twist-boat) behave soft and labile in conformation. The soft parts of the molecules are certainly controlled by the steric requirements of the ring substituents and by the crystal packing with its interplay of intra- and intermolecular forces. In the title compound 6α-acetoxygedunin such forces involve only several quite weak intra- and inter-molecular C—H···O interactions, which are listed in Table 1. For a packing diagram of 6α-acetoxygedunin, see Fig. 5.

### Experimental

Air-dried root bark of *Aglaia elaeagnoidea* (28 g) collected at the shore near Port Douglas, Queensland, northern Australia, was ground and extracted with MeOH at room temperature for 3 days, filtered and concentrated. The CHCl_3_ fraction (1.2 g) of the aqueous residue was roughly separated by column chromatography (Merck Si gel 60, 35–70 mesh) eluted initially with hexane enriched with EtOAc, followed by an increasing amount of MeOH in EtOAc and finally with MeOH. The fraction eluted with 50% EtOAc in hexane was further separated by repeated preparative MPLC (400 *x* 40 mm column, Merck LiChroprep silica 60, 25–40 µm, UV detection at 229 and 254 nm) using 5% 2-propanol in hexane yielding 15 mg of impure 6α-acetoxygedunin from which 3.8 mg of crystals could be obtained.

The resonances of the ^1^H and ^13^C NMR spectra of the title compound were assigned by two-dimensional NMR (H/H COSY, NOESY, HMBC, HMQC) and relevant literature for 11β-acetoxygedunin (Connolly *et al.*, 1966) and gedunin (Taylor, 1974). ^1^H NMR (400 MHz, CDCl_3_, δ/p.p.m.): 7.41 (d, 1H, J= 1.3 Hz, 21-H), 7.41 (d, 1H, J = 1.3 Hz, 23-H), 7.07 (d, 1H, J = 10.1 Hz, 1-H), 6.33 (t, 1H, J = 1.3 Hz, 22-H), 5.94 (d, 1H, J = 10.1 Hz, 2-H), 5.61 (s, 1H, 17-H), 5.27 (dd, 1H, J = 12.4 and 2.4 Hz, 6-H), 4.89 (d, 1H, J = 2.4 Hz, 7-H), 3.61 (s, 1H, 15-H), 2.53 (m, 1H, 9-H), 2.52 (d, 1H, J = 12.4 Hz, 5-H), 2.15 (s, 3H, 7-OAc), 2.03 (s, 3H, 6-OAc), 1.60, 1.35, 1.30, and 1.10 (m, 1H each, 11-H2 and 12-H2), 1.27 (s, 3H, 26-H3), 1.26 (s, 3H, 24-H3), 1.24 (s, 3H, 18-H3), 1.21 (s, 3H, 19-H3), 1.17 (s, 3H, 25-H3). ^13^C NMR (CDCl_3_, δ/p.p.m.): 204.1 (s, C-3), 170.1 and 170.0 (s, 6- and 7-acetyl CO), 167.1 (s, C-16), 156.2 (d, C-1), 143.1 (d, C-23), 141.2 (d, C-21), 126.6 (d, C-2), 120.3 (s, C-20), 109.8 (d, C-22), 78.1 (d, C-17), 72.6 (d, C-7), 69.7 (d, C-6), 69.5 (s, C-14), 56.2 (d, C-15), 47.8 (d, C-5), 44.9 (s, C-4), 43.1 (s, C-8), 40.6 (s, C10), 48.8 (s, C-13), 38.4 (d, C-9), 31.6 (q, C-24), 25.9 (t, C-12), 21.4 (q, C-19), 21.2 (q, 6-acetyl CH_3_), 20.9 (q, 7-acetyl CH_3_), 20.2 (q, C-25), 18.1 (q, C-26), 17.9 (q, C-18), 15.0 (t, C-11).

### Refinement

All C-bound H atoms were placed in calculated positions (C—H = 0.95–1.00 Å) and thereafter treated as riding. A torsional parameter was refined for each methyl group. *U*_iso_(H) = 1.2*U*_eq_(C) and *U*_iso_(H) = 1.5*U*_eq_(C_methyl_) were applied. The absolute structure could not be determined from the X-ray analysis, but it is known from earlier work on related compounds (*e.g.* Sutherland *et al.*, 1962). Friedel pairs were therefore merged before final refinement.

### Crystal data

**Table:**

C_30_H_36_O_9_	*F*(000) = 1152
*M**_r_* = 540.59	*D*_x_ = 1.295 Mg m^−^^3^
Orthorhombic, *P*2_1_2_1_2_1_	Mo *K*α radiation, λ = 0.71073 Å
Hall symbol: P 2ac 2ab	Cell parameters from 852 reflections
*a* = 6.475 (2) Å	θ = 2.4–29.8°
*b* = 14.914 (5) Å	µ = 0.10 mm^−^^1^
*c* = 28.713 (9) Å	*T* = 173 K
*V* = 2772.8 (15) Å^3^	Prism, colourless
*Z* = 4	0.62 × 0.40 × 0.25 mm

### Data collection

**Table:**

Bruker SMART APEX CCD diffractometer	4509 independent reflections
Radiation source: fine-focus sealed tube	4077 reflections with *I* > 2σ(*I*)
graphite	*R*_int_ = 0.031
ω scans	θ_max_ = 30.0°, θ_min_ = 2.5°
Absorption correction: multi-scan (*SADABS*; Bruker, 2003)	*h* = −9→9
*T*_min_ = 0.89, *T*_max_ = 0.98	*k* = −20→20
39048 measured reflections	*l* = −40→40

### Refinement

**Table:**

Refinement on *F*^2^	Primary atom site location: structure-invariant direct methods
Least-squares matrix: full	Secondary atom site location: difference Fourier map
*R*[*F*^2^ > 2σ(*F*^2^)] = 0.037	Hydrogen site location: inferred from neighbouring sites
*wR*(*F*^2^) = 0.099	H-atom parameters constrained
*S* = 1.04	*w* = 1/[σ^2^(*F*_o_^2^) + (0.0617*P*)^2^ + 0.3403*P*] where *P* = (*F*_o_^2^ + 2*F*_c_^2^)/3
4509 reflections	(Δ/σ)_max_ < 0.001
359 parameters	Δρ_max_ = 0.26 e Å^−^^3^
0 restraints	Δρ_min_ = −0.24 e Å^−^^3^

### Special details

**Table:**

Geometry. All e.s.d.'s (except the e.s.d. in the dihedral angle between two l.s. planes) are estimated using the full covariance matrix. The cell e.s.d.'s are taken into account individually in the estimation of e.s.d.'s in distances, angles and torsion angles; correlations between e.s.d.'s in cell parameters are only used when they are defined by crystal symmetry. An approximate (isotropic) treatment of cell e.s.d.'s is used for estimating e.s.d.'s involving l.s. planes.
Refinement. Refinement of *F*^2^ against ALL reflections. The weighted *R*-factor *wR* and goodness of fit *S* are based on *F*^2^, conventional *R*-factors *R* are based on *F*, with *F* set to zero for negative *F*^2^. The threshold expression of *F*^2^ > σ(*F*^2^) is used only for calculating *R*-factors(gt) *etc*. and is not relevant to the choice of reflections for refinement. *R*-factors based on *F*^2^ are statistically about twice as large as those based on *F*, and *R*- factors based on ALL data will be even larger.

### Fractional atomic coordinates and isotropic or equivalent isotropic displacement parameters (Å^2^)

**Table:**

	*x*	*y*	*z*	*U*_iso_*/*U*_eq_	
O1	0.0587 (3)	0.73999 (11)	0.23019 (6)	0.0535 (4)	
O2	0.3222 (2)	0.68452 (7)	0.40284 (4)	0.0308 (3)	
O3	0.21379 (17)	0.50631 (7)	0.42004 (3)	0.0218 (2)	
O4	0.67376 (18)	0.33557 (9)	0.41784 (4)	0.0303 (3)	
O5	0.3916 (2)	0.20217 (8)	0.45502 (4)	0.0311 (3)	
O6	0.3600 (2)	0.29114 (9)	0.51630 (4)	0.0371 (3)	
O7	0.3049 (7)	−0.02998 (11)	0.35841 (9)	0.1021 (11)	
O8	0.6555 (3)	0.70255 (12)	0.42549 (6)	0.0546 (4)	
O9	0.3152 (3)	0.55979 (10)	0.49051 (4)	0.0413 (3)	
C1	0.1155 (3)	0.51480 (13)	0.26444 (5)	0.0295 (3)	
H1	0.0712	0.4558	0.2569	0.035*	
C2	0.0178 (3)	0.58569 (14)	0.24571 (6)	0.0342 (4)	
H2	−0.0937	0.5765	0.2247	0.041*	
C3	0.0830 (3)	0.67781 (14)	0.25763 (6)	0.0354 (4)	
C4	0.1727 (3)	0.69563 (11)	0.30666 (6)	0.0289 (3)	
C5	0.2297 (2)	0.60496 (10)	0.33182 (5)	0.0220 (3)	
H5	0.0998	0.5837	0.3471	0.026*	
C6	0.3893 (2)	0.61484 (10)	0.37098 (5)	0.0230 (3)	
H6	0.5258	0.6318	0.3573	0.028*	
C7	0.4135 (2)	0.52809 (10)	0.39934 (5)	0.0210 (3)	
H7	0.5176	0.5376	0.4246	0.025*	
C8	0.4803 (2)	0.44830 (10)	0.36887 (5)	0.0198 (3)	
C9	0.3279 (2)	0.43982 (10)	0.32698 (5)	0.0208 (3)	
H9	0.1901	0.4274	0.3413	0.025*	
C10	0.2973 (2)	0.52800 (10)	0.29783 (5)	0.0217 (3)	
C11	0.3781 (3)	0.35593 (11)	0.29761 (5)	0.0302 (3)	
H11A	0.4995	0.3692	0.2778	0.036*	
H11B	0.2600	0.3436	0.2767	0.036*	
C12	0.4241 (3)	0.27008 (11)	0.32647 (6)	0.0300 (3)	
H12A	0.3503	0.2188	0.3122	0.036*	
H12B	0.5739	0.2572	0.3249	0.036*	
C13	0.3597 (2)	0.27762 (10)	0.37810 (5)	0.0230 (3)	
C14	0.4749 (2)	0.35925 (10)	0.39801 (5)	0.0210 (3)	
C15	0.5091 (2)	0.35775 (11)	0.44934 (5)	0.0248 (3)	
H15	0.5181	0.4171	0.4654	0.030*	
C16	0.4171 (3)	0.28141 (11)	0.47670 (5)	0.0268 (3)	
C17	0.4394 (3)	0.19354 (11)	0.40509 (6)	0.0325 (4)	
H17	0.5926	0.1892	0.4012	0.039*	
C18	0.1228 (2)	0.28506 (11)	0.38491 (5)	0.0254 (3)	
H18A	0.0824	0.3484	0.3851	0.038*	
H18B	0.0840	0.2574	0.4146	0.038*	
H18C	0.0522	0.2541	0.3594	0.038*	
C19	0.4839 (3)	0.55432 (12)	0.26637 (5)	0.0286 (3)	
H19A	0.5308	0.5016	0.2490	0.043*	
H19B	0.5969	0.5767	0.2859	0.043*	
H19C	0.4413	0.6012	0.2445	0.043*	
C20	0.3434 (4)	0.10558 (12)	0.39106 (7)	0.0455 (5)	
C21	0.4335 (7)	0.04307 (15)	0.36416 (10)	0.0767 (11)	
H21	0.5673	0.0484	0.3509	0.092*	
C22	0.1459 (5)	0.06906 (14)	0.40432 (10)	0.0588 (7)	
H22	0.0451	0.0969	0.4235	0.071*	
C23	0.1322 (8)	−0.01205 (18)	0.38421 (13)	0.0876 (13)	
H23	0.0181	−0.0516	0.3875	0.105*	
C24	−0.0104 (3)	0.74131 (13)	0.33278 (7)	0.0399 (4)	
H24A	−0.0409	0.7993	0.3182	0.060*	
H24B	0.0274	0.7507	0.3655	0.060*	
H24C	−0.1327	0.7027	0.3311	0.060*	
C25	0.3518 (3)	0.76299 (12)	0.30263 (7)	0.0367 (4)	
H25A	0.3057	0.8158	0.2852	0.055*	
H25B	0.4678	0.7348	0.2863	0.055*	
H25C	0.3961	0.7813	0.3339	0.055*	
C26	0.7086 (2)	0.46280 (12)	0.35367 (6)	0.0269 (3)	
H26A	0.7211	0.5204	0.3375	0.040*	
H26B	0.7507	0.4142	0.3327	0.040*	
H26C	0.7978	0.4629	0.3813	0.040*	
C27	0.4760 (4)	0.72270 (14)	0.42919 (7)	0.0419 (5)	
C28	0.3892 (6)	0.79118 (17)	0.46226 (9)	0.0655 (8)	
H28A	0.4966	0.8349	0.4702	0.098*	
H28B	0.3415	0.7612	0.4907	0.098*	
H28C	0.2729	0.8221	0.4475	0.098*	
C29	0.1864 (3)	0.52448 (11)	0.46639 (5)	0.0268 (3)	
C30	−0.0241 (3)	0.49403 (14)	0.48189 (6)	0.0382 (4)	
H30A	−0.0316	0.4956	0.5160	0.057*	
H30B	−0.0485	0.4327	0.4710	0.057*	
H30C	−0.1294	0.5340	0.4688	0.057*	

### Atomic displacement parameters (Å^2^)

**Table:**

	*U*^11^	*U*^22^	*U*^33^	*U*^12^	*U*^13^	*U*^23^
O1	0.0596 (10)	0.0553 (9)	0.0454 (8)	0.0088 (8)	−0.0091 (7)	0.0263 (7)
O2	0.0402 (7)	0.0240 (5)	0.0281 (5)	−0.0007 (5)	0.0018 (5)	−0.0018 (4)
O3	0.0219 (5)	0.0257 (5)	0.0177 (4)	−0.0016 (4)	0.0032 (4)	0.0006 (4)
O4	0.0194 (5)	0.0432 (6)	0.0284 (5)	0.0036 (5)	0.0000 (4)	0.0133 (5)
O5	0.0357 (6)	0.0287 (6)	0.0289 (6)	−0.0005 (5)	−0.0027 (5)	0.0088 (5)
O6	0.0407 (7)	0.0463 (7)	0.0242 (5)	−0.0066 (6)	0.0035 (5)	0.0092 (5)
O7	0.200 (3)	0.0281 (8)	0.0781 (14)	0.0038 (15)	−0.016 (2)	−0.0129 (8)
O8	0.0467 (9)	0.0676 (10)	0.0496 (8)	−0.0210 (8)	−0.0039 (7)	−0.0149 (8)
O9	0.0463 (8)	0.0528 (8)	0.0247 (5)	−0.0105 (7)	0.0018 (6)	−0.0083 (6)
C1	0.0263 (8)	0.0408 (9)	0.0213 (6)	−0.0019 (7)	−0.0024 (6)	0.0031 (6)
C2	0.0247 (8)	0.0528 (10)	0.0251 (7)	0.0013 (8)	−0.0037 (6)	0.0097 (7)
C3	0.0259 (8)	0.0483 (10)	0.0319 (8)	0.0067 (8)	0.0012 (7)	0.0147 (7)
C4	0.0283 (8)	0.0298 (7)	0.0285 (7)	0.0048 (7)	0.0039 (6)	0.0101 (6)
C5	0.0198 (6)	0.0259 (7)	0.0202 (6)	0.0005 (5)	0.0021 (5)	0.0052 (5)
C6	0.0242 (7)	0.0232 (7)	0.0215 (6)	−0.0016 (6)	0.0023 (6)	0.0006 (5)
C7	0.0198 (6)	0.0250 (7)	0.0182 (6)	−0.0022 (5)	0.0004 (5)	0.0015 (5)
C8	0.0178 (6)	0.0238 (6)	0.0179 (6)	0.0006 (5)	0.0002 (5)	0.0035 (5)
C9	0.0211 (6)	0.0248 (6)	0.0166 (6)	−0.0007 (6)	0.0007 (5)	0.0023 (5)
C10	0.0205 (6)	0.0278 (7)	0.0169 (6)	0.0005 (6)	0.0011 (5)	0.0037 (5)
C11	0.0451 (10)	0.0276 (7)	0.0178 (6)	0.0009 (7)	0.0028 (7)	−0.0010 (6)
C12	0.0372 (9)	0.0284 (7)	0.0244 (7)	0.0084 (7)	0.0042 (6)	−0.0021 (6)
C13	0.0253 (7)	0.0213 (6)	0.0224 (6)	0.0049 (6)	0.0016 (5)	0.0015 (5)
C14	0.0184 (6)	0.0259 (7)	0.0187 (6)	0.0027 (5)	0.0010 (5)	0.0037 (5)
C15	0.0224 (7)	0.0308 (7)	0.0212 (6)	−0.0018 (6)	−0.0020 (6)	0.0065 (5)
C16	0.0229 (7)	0.0325 (8)	0.0249 (7)	−0.0006 (6)	−0.0026 (6)	0.0093 (6)
C17	0.0406 (9)	0.0257 (7)	0.0311 (8)	0.0103 (7)	0.0012 (7)	0.0049 (6)
C18	0.0242 (7)	0.0258 (7)	0.0262 (7)	−0.0016 (6)	−0.0013 (6)	0.0031 (6)
C19	0.0257 (7)	0.0383 (8)	0.0217 (6)	0.0022 (7)	0.0056 (6)	0.0082 (6)
C20	0.0767 (16)	0.0233 (8)	0.0365 (9)	0.0091 (10)	−0.0058 (10)	0.0034 (7)
C21	0.138 (3)	0.0313 (10)	0.0610 (15)	0.0238 (15)	0.0115 (19)	−0.0033 (10)
C22	0.0819 (18)	0.0284 (9)	0.0661 (14)	−0.0109 (11)	−0.0189 (14)	0.0047 (9)
C23	0.142 (4)	0.0335 (12)	0.087 (2)	−0.0168 (18)	−0.032 (3)	−0.0012 (13)
C24	0.0366 (9)	0.0372 (9)	0.0458 (10)	0.0111 (8)	0.0102 (8)	0.0104 (8)
C25	0.0387 (9)	0.0318 (8)	0.0397 (9)	−0.0024 (7)	0.0049 (8)	0.0140 (7)
C26	0.0180 (6)	0.0364 (8)	0.0263 (7)	0.0001 (6)	0.0030 (6)	0.0083 (6)
C27	0.0607 (13)	0.0341 (9)	0.0309 (8)	−0.0134 (9)	−0.0003 (9)	−0.0065 (7)
C28	0.095 (2)	0.0476 (13)	0.0538 (13)	−0.0037 (15)	−0.0014 (14)	−0.0251 (11)
C29	0.0343 (8)	0.0266 (7)	0.0195 (6)	−0.0010 (7)	0.0055 (6)	−0.0014 (5)
C30	0.0412 (10)	0.0434 (10)	0.0299 (8)	−0.0086 (8)	0.0164 (7)	−0.0055 (7)

### Geometric parameters (Å, °)

**Table:**

O1—C3	1.227 (2)	C12—C13	1.544 (2)
O2—C27	1.374 (3)	C12—H12A	0.9900
O2—C6	1.4510 (18)	C12—H12B	0.9900
O3—C29	1.3697 (18)	C13—C14	1.538 (2)
O3—C7	1.4600 (18)	C13—C18	1.551 (2)
O4—C15	1.436 (2)	C13—C17	1.562 (2)
O4—C14	1.4517 (18)	C14—C15	1.491 (2)
O5—C16	1.346 (2)	C15—C16	1.506 (2)
O5—C17	1.472 (2)	C15—H15	1.0000
O6—C16	1.204 (2)	C17—C20	1.507 (3)
O7—C23	1.368 (6)	C17—H17	1.0000
O7—C21	1.381 (5)	C18—H18A	0.9800
O8—C27	1.205 (3)	C18—H18B	0.9800
O9—C29	1.205 (2)	C18—H18C	0.9800
C1—C2	1.344 (2)	C19—H19A	0.9800
C1—C10	1.531 (2)	C19—H19B	0.9800
C1—H1	0.9500	C19—H19C	0.9800
C2—C3	1.478 (3)	C20—C21	1.344 (3)
C2—H2	0.9500	C20—C22	1.441 (4)
C3—C4	1.546 (3)	C21—H21	0.9500
C4—C25	1.539 (3)	C22—C23	1.343 (4)
C4—C24	1.560 (3)	C22—H22	0.9500
C4—C5	1.577 (2)	C23—H23	0.9500
C5—C6	1.534 (2)	C24—H24A	0.9800
C5—C10	1.569 (2)	C24—H24B	0.9800
C5—H5	1.0000	C24—H24C	0.9800
C6—C7	1.537 (2)	C25—H25A	0.9800
C6—H6	1.0000	C25—H25B	0.9800
C7—C8	1.539 (2)	C25—H25C	0.9800
C7—H7	1.0000	C26—H26A	0.9800
C8—C26	1.557 (2)	C26—H26B	0.9800
C8—C9	1.561 (2)	C26—H26C	0.9800
C8—C14	1.570 (2)	C27—C28	1.504 (3)
C9—C11	1.544 (2)	C28—H28A	0.9800
C9—C10	1.571 (2)	C28—H28B	0.9800
C9—H9	1.0000	C28—H28C	0.9800
C10—C19	1.559 (2)	C29—C30	1.504 (3)
C11—C12	1.554 (2)	C30—H30A	0.9800
C11—H11A	0.9900	C30—H30B	0.9800
C11—H11B	0.9900	C30—H30C	0.9800
			
C27—O2—C6	115.28 (15)	C15—C14—C8	122.44 (13)
C29—O3—C7	117.80 (12)	C13—C14—C8	118.83 (12)
C15—O4—C14	62.14 (9)	O4—C15—C14	59.43 (9)
C16—O5—C17	120.09 (12)	O4—C15—C16	116.61 (14)
C23—O7—C21	105.9 (2)	C14—C15—C16	117.91 (14)
C2—C1—C10	120.74 (16)	O4—C15—H15	116.8
C2—C1—H1	119.6	C14—C15—H15	116.8
C10—C1—H1	119.6	C16—C15—H15	116.8
C1—C2—C3	120.26 (16)	O6—C16—O5	120.32 (15)
C1—C2—H2	119.9	O6—C16—C15	121.52 (16)
C3—C2—H2	119.9	O5—C16—C15	118.11 (14)
O1—C3—C2	121.14 (18)	O5—C17—C20	104.45 (14)
O1—C3—C4	120.21 (19)	O5—C17—C13	110.09 (13)
C2—C3—C4	118.58 (14)	C20—C17—C13	115.47 (15)
C25—C4—C3	109.08 (14)	O5—C17—H17	108.9
C25—C4—C24	108.90 (16)	C20—C17—H17	108.9
C3—C4—C24	103.16 (15)	C13—C17—H17	108.9
C25—C4—C5	114.70 (14)	C13—C18—H18A	109.5
C3—C4—C5	110.96 (14)	C13—C18—H18B	109.5
C24—C4—C5	109.39 (13)	H18A—C18—H18B	109.5
C6—C5—C10	109.77 (12)	C13—C18—H18C	109.5
C6—C5—C4	114.27 (13)	H18A—C18—H18C	109.5
C10—C5—C4	114.05 (12)	H18B—C18—H18C	109.5
C6—C5—H5	106.0	C10—C19—H19A	109.5
C10—C5—H5	106.0	C10—C19—H19B	109.5
C4—C5—H5	106.0	H19A—C19—H19B	109.5
O2—C6—C5	109.22 (12)	C10—C19—H19C	109.5
O2—C6—C7	107.43 (12)	H19A—C19—H19C	109.5
C5—C6—C7	112.10 (12)	H19B—C19—H19C	109.5
O2—C6—H6	109.3	C21—C20—C22	106.0 (3)
C5—C6—H6	109.3	C21—C20—C17	125.3 (3)
C7—C6—H6	109.3	C22—C20—C17	128.7 (2)
O3—C7—C6	108.21 (12)	C20—C21—O7	110.8 (4)
O3—C7—C8	107.95 (11)	C20—C21—H21	124.6
C6—C7—C8	112.24 (12)	O7—C21—H21	124.6
O3—C7—H7	109.5	C23—C22—C20	106.6 (3)
C6—C7—H7	109.5	C23—C22—H22	126.7
C8—C7—H7	109.5	C20—C22—H22	126.7
C7—C8—C26	108.58 (13)	C22—C23—O7	110.8 (4)
C7—C8—C9	108.85 (12)	C22—C23—H23	124.6
C26—C8—C9	113.32 (11)	O7—C23—H23	124.6
C7—C8—C14	110.18 (11)	C4—C24—H24A	109.5
C26—C8—C14	106.74 (12)	C4—C24—H24B	109.5
C9—C8—C14	109.15 (12)	H24A—C24—H24B	109.5
C11—C9—C8	110.69 (12)	C4—C24—H24C	109.5
C11—C9—C10	114.45 (11)	H24A—C24—H24C	109.5
C8—C9—C10	114.98 (12)	H24B—C24—H24C	109.5
C11—C9—H9	105.2	C4—C25—H25A	109.5
C8—C9—H9	105.2	C4—C25—H25B	109.5
C10—C9—H9	105.2	H25A—C25—H25B	109.5
C1—C10—C19	105.40 (12)	C4—C25—H25C	109.5
C1—C10—C5	105.61 (13)	H25A—C25—H25C	109.5
C19—C10—C5	113.12 (13)	H25B—C25—H25C	109.5
C1—C10—C9	108.83 (13)	C8—C26—H26A	109.5
C19—C10—C9	114.95 (13)	C8—C26—H26B	109.5
C5—C10—C9	108.42 (11)	H26A—C26—H26B	109.5
C9—C11—C12	114.64 (12)	C8—C26—H26C	109.5
C9—C11—H11A	108.6	H26A—C26—H26C	109.5
C12—C11—H11A	108.6	H26B—C26—H26C	109.5
C9—C11—H11B	108.6	O8—C27—O2	123.16 (18)
C12—C11—H11B	108.6	O8—C27—C28	125.8 (2)
H11A—C11—H11B	107.6	O2—C27—C28	111.0 (2)
C13—C12—C11	113.60 (13)	C27—C28—H28A	109.5
C13—C12—H12A	108.8	C27—C28—H28B	109.5
C11—C12—H12A	108.8	H28A—C28—H28B	109.5
C13—C12—H12B	108.8	C27—C28—H28C	109.5
C11—C12—H12B	108.8	H28A—C28—H28C	109.5
H12A—C12—H12B	107.7	H28B—C28—H28C	109.5
C14—C13—C12	106.49 (13)	O9—C29—O3	123.71 (16)
C14—C13—C18	112.10 (13)	O9—C29—C30	126.10 (15)
C12—C13—C18	113.16 (13)	O3—C29—C30	110.19 (14)
C14—C13—C17	106.91 (13)	C29—C30—H30A	109.5
C12—C13—C17	109.19 (13)	C29—C30—H30B	109.5
C18—C13—C17	108.77 (14)	H30A—C30—H30B	109.5
O4—C14—C15	58.43 (9)	C29—C30—H30C	109.5
O4—C14—C13	112.54 (12)	H30A—C30—H30C	109.5
C15—C14—C13	115.33 (12)	H30B—C30—H30C	109.5
O4—C14—C8	113.27 (12)		
			
C10—C1—C2—C3	−0.8 (3)	C11—C12—C13—C18	66.3 (2)
C1—C2—C3—O1	151.7 (2)	C11—C12—C13—C17	−172.40 (15)
C1—C2—C3—C4	−31.5 (3)	C15—O4—C14—C13	−106.78 (14)
O1—C3—C4—C25	−41.8 (2)	C15—O4—C14—C8	114.90 (14)
C2—C3—C4—C25	141.42 (17)	C12—C13—C14—O4	−90.70 (14)
O1—C3—C4—C24	73.9 (2)	C18—C13—C14—O4	145.05 (12)
C2—C3—C4—C24	−102.93 (18)	C17—C13—C14—O4	25.94 (16)
O1—C3—C4—C5	−169.06 (17)	C12—C13—C14—C15	−155.18 (13)
C2—C3—C4—C5	14.1 (2)	C18—C13—C14—C15	80.56 (16)
C25—C4—C5—C6	33.94 (19)	C17—C13—C14—C15	−38.54 (18)
C3—C4—C5—C6	158.10 (13)	C12—C13—C14—C8	45.10 (17)
C24—C4—C5—C6	−88.73 (17)	C18—C13—C14—C8	−79.16 (16)
C25—C4—C5—C10	−93.47 (17)	C17—C13—C14—C8	161.74 (13)
C3—C4—C5—C10	30.69 (18)	C7—C8—C14—O4	−95.19 (14)
C24—C4—C5—C10	143.86 (15)	C26—C8—C14—O4	22.52 (16)
C27—O2—C6—C5	−157.85 (14)	C9—C8—C14—O4	145.34 (12)
C27—O2—C6—C7	80.34 (16)	C7—C8—C14—C15	−28.89 (19)
C10—C5—C6—O2	−178.48 (11)	C26—C8—C14—C15	88.82 (16)
C4—C5—C6—O2	51.94 (16)	C9—C8—C14—C15	−148.36 (14)
C10—C5—C6—C7	−59.54 (15)	C7—C8—C14—C13	129.32 (13)
C4—C5—C6—C7	170.89 (12)	C26—C8—C14—C13	−112.97 (14)
C29—O3—C7—C6	−103.01 (14)	C9—C8—C14—C13	9.85 (17)
C29—O3—C7—C8	135.29 (13)	C14—O4—C15—C16	108.19 (15)
O2—C6—C7—O3	59.79 (15)	C13—C14—C15—O4	101.94 (14)
C5—C6—C7—O3	−60.21 (15)	C8—C14—C15—O4	−99.15 (15)
O2—C6—C7—C8	178.81 (12)	O4—C14—C15—C16	−106.01 (16)
C5—C6—C7—C8	58.81 (16)	C13—C14—C15—C16	−4.1 (2)
O3—C7—C8—C26	−170.06 (11)	C8—C14—C15—C16	154.85 (14)
C6—C7—C8—C26	70.76 (15)	C17—O5—C16—O6	172.96 (16)
O3—C7—C8—C9	66.16 (14)	C17—O5—C16—C15	−4.3 (2)
C6—C7—C8—C9	−53.01 (16)	O4—C15—C16—O6	143.88 (16)
O3—C7—C8—C14	−53.49 (14)	C14—C15—C16—O6	−148.35 (16)
C6—C7—C8—C14	−172.67 (12)	O4—C15—C16—O5	−38.9 (2)
C7—C8—C9—C11	−175.38 (12)	C14—C15—C16—O5	28.8 (2)
C26—C8—C9—C11	63.71 (16)	C16—O5—C17—C20	−166.21 (16)
C14—C8—C9—C11	−55.09 (15)	C16—O5—C17—C13	−41.7 (2)
C7—C8—C9—C10	53.01 (15)	C14—C13—C17—O5	61.47 (17)
C26—C8—C9—C10	−67.90 (16)	C12—C13—C17—O5	176.30 (14)
C14—C8—C9—C10	173.30 (12)	C18—C13—C17—O5	−59.77 (18)
C2—C1—C10—C19	−75.97 (19)	C14—C13—C17—C20	179.41 (16)
C2—C1—C10—C5	44.02 (19)	C12—C13—C17—C20	−65.8 (2)
C2—C1—C10—C9	160.24 (15)	C18—C13—C17—C20	58.2 (2)
C6—C5—C10—C1	172.32 (12)	O5—C17—C20—C21	−135.9 (2)
C4—C5—C10—C1	−57.99 (16)	C13—C17—C20—C21	103.1 (3)
C6—C5—C10—C19	−72.91 (15)	O5—C17—C20—C22	41.4 (3)
C4—C5—C10—C19	56.78 (17)	C13—C17—C20—C22	−79.6 (2)
C6—C5—C10—C9	55.81 (15)	C22—C20—C21—O7	1.5 (3)
C4—C5—C10—C9	−174.49 (12)	C17—C20—C21—O7	179.3 (2)
C11—C9—C10—C1	61.00 (17)	C23—O7—C21—C20	−2.0 (3)
C8—C9—C10—C1	−169.21 (12)	C21—C20—C22—C23	−0.3 (3)
C11—C9—C10—C19	−56.92 (18)	C17—C20—C22—C23	−178.1 (2)
C8—C9—C10—C19	72.88 (16)	C20—C22—C23—O7	−0.9 (3)
C11—C9—C10—C5	175.40 (13)	C21—O7—C23—C22	1.8 (4)
C8—C9—C10—C5	−54.81 (16)	C6—O2—C27—O8	2.3 (3)
C8—C9—C11—C12	43.91 (19)	C6—O2—C27—C28	−177.72 (17)
C10—C9—C11—C12	175.79 (14)	C7—O3—C29—O9	2.3 (2)
C9—C11—C12—C13	14.2 (2)	C7—O3—C29—C30	−177.55 (13)
C11—C12—C13—C14	−57.29 (18)		

### Hydrogen-bond geometry (Å, °)

**Table:**

*D*—H···*A*	*D*—H	H···*A*	*D*···*A*	*D*—H···*A*
C11—H11B···O1^i^	0.99	2.59	3.410 (3)	141
C18—H18C···O1^i^	0.98	2.68	3.571 (2)	152
C18—H18B···O6^ii^	0.98	2.56	3.497 (2)	160
C22—H22···O6^ii^	0.95	2.69	3.601 (3)	162
C24—H24B···O2	0.98	2.40	3.066 (3)	125
C25—H25C···O2	0.98	2.50	3.112 (2)	121
C5—H5···O3	1.00	2.50	2.931 (2)	105
C9—H9···O3	1.00	2.55	2.944 (2)	103
C18—H18B···O5	0.98	2.45	2.934 (2)	110
C7—H7···O9	1.00	2.33	2.735 (2)	103

Symmetry codes: (i) −*x*, *y*−1/2, −*z*+1/2; (ii) *x*−1/2, −*y*+1/2, −*z*+1.
